# Corrosion Protective Coating Based on Chemically Cross-Linked Particles of Few-Layer Graphene

**DOI:** 10.3390/nano15241841

**Published:** 2025-12-05

**Authors:** Aleksei Vozniakovskii, Alexander Voznyakovskii, Anna Neverovskaya, Nikita Podlozhnyuk, Sergey Kidalov, Evgeny Auchynnikau

**Affiliations:** 1 Ioffe Institute, 194021 Saint-Petersburg, Russia; 2 Institute for Synthetic Rubber, 198035 Saint-Petersburg, Russia; 3 Department of Logistics and Management Methods, Yanka Kupala State University of Grodno, 230023 Grodno, Belarus

**Keywords:** few-layer graphene, coating, corrosion, self-propagating high-temperature synthesis, chemical cross-linking

## Abstract

Coatings based on graphene nanostructures represent one of the most promising solutions for protecting metals from corrosion. However, their application remains unprofitable due to the high production costs, which are caused by the imperfections in graphene nanostructures synthesis methods. Therefore, this work utilized few-layer graphene particles synthesized via self-propagating high-temperature synthesis for coating fabrication. The effectiveness of these coatings in protecting metals against corrosion was tested in a salt spray chamber. It was found that the synthesized coatings provide excellent protection for the steel substrate against corrosion, and their effectiveness is significantly higher than that of polymer coatings based on epoxy resin. A hypothesis was proposed to explain the high efficiency of the coatings based on few-layer graphene particles. This is attributed to their low defect density (absence of Stone-Wales defects in their structure) and the presence of multiple layers, which enhances the barrier effect.

## 1. Introduction

For thousands of years, since the dawn of active metal usage, humanity has engaged in a constant battle against corrosion. One of the most effective methods of corrosion protection is the application of various coatings [1]. A wide range of materials is used as the basis for such coatings: metals (such as zinc or lead) [2,3,4], polymers [5,6,7,8,9], and ceramics [10,11]. However, despite the abundance of anti-corrosion coatings, corrosion remains a massive problem. According to various estimates, the annual global economic cost of corrosion reaches up to 2.5 trillion US dollars [12]. Consequently, researchers are continuously seeking new materials and approaches to create more advanced anti-corrosion coatings.

Graphene nanostructures (GNS) are materials consisting of no more than 10 layers of graphene [13]. Due to their dense structure, dictated by the size of the carbon hexagons, GNS create a so-called “barrier effect”. This effect prevents molecules of water and oxygen from penetrating through the GNS and initiating the corrosion process [14]. Therefore, GNS are widely used in the creation of anti-corrosion coatings [15,16,17], both on their own and as a modifying additive, for example, in the creation of polymer composite coatings [18,19]. However, despite a significant number of studies reporting high anti-corrosion efficiency of GNS-based coatings [20,21], their widespread practical application has not yet been realized.

The primary reason for the lack of mass adoption of GNS-based coatings is their high cost. This, in turn, is caused by the imperfections in GNS synthesis methodologies, both “bottom-up” [22,23] and “top-down” [24]. These methods are unable to produce GNS in large volumes with high quality and at an acceptable cost. Reviews [25,20,26] have noted that the presence of various structural defects significantly reduces the barrier effectiveness of GNS, leading to diminished anti-corrosion performance, and have also highlighted the limitations of existing GNS synthesis methods.

Therefore, for the implementation of GNS-based anti-corrosion coatings, a high-throughput methodology is required—one that enables the synthesis of large volumes of GNS with high quality and low cost.

To address this need, in our previous work [27], we demonstrated the possibility of synthesizing few-layer graphene (FLG, no more than 5 layers) from cyclic biopolymers [28] under conditions of a Self-propagating High-temperature Synthesis (SHS) process. This method allows for the synthesis of large material volumes and yields FLG free of Stone-Wales defects in its structure [29]. In our previous studies, we demonstrated that the synthesized few-layer graphene (FLG) is an effective modifying additive for creating polymer composites aimed at enhancing a combination of strength, thermophysical, and tribological properties [30,31].

This work, for the first time, utilizes FLG particles synthesized via a SHS process to create a coating through chemical cross-linking of the particles. It also presents the initial results of their effectiveness in protecting metals from corrosion.

## 2. Materials and Methods

### 2.1. Initial Material for Synthesis of FLG Coatings

The coating was synthesized using few-layer graphene (FLG) with no more than five layers, which was produced via a self-propagating high-temperature synthesis (SHS) process from glucose. For the FLG synthesis, glucose was mixed with ammonium nitrate in a 1:1 mass ratio using a tumbler mixer. The resulting mixture was placed in a reactor and heated to 250 °C, which corresponds to the ignition temperature of the SHS process. The initiation of the SHS process was marked by the onset of vigorous gas evolution, and its completion was determined by the cessation of gas release.

Upon completion of the synthesis, the FLG powder was washed with deionized water and dried in an oven at 110 °C until a constant mass was achieved. The detailed synthesis procedure is described in reference [28]. Using a chemical methodology for the quantitative detection of Stone–Wales defects [29], it was confirmed that the synthesized FLG contained no such defects within the detection limits of the method.

### 2.2. Coating Synthesis Methodology

Graphene nanostructures (GNS) feature various functional groups, such as -OH and -COOH, at the edges of their graphene sheets [32,33]. These same surface groups are also present in the few-layer graphene (FLG) used in this study as the starting material for coating synthesis. Their presence enables the chemical cross-linking of FLG particles via these specific functional groups, which was performed using the Chugaev–Tseretitinov method [34].

**Figure 1:**
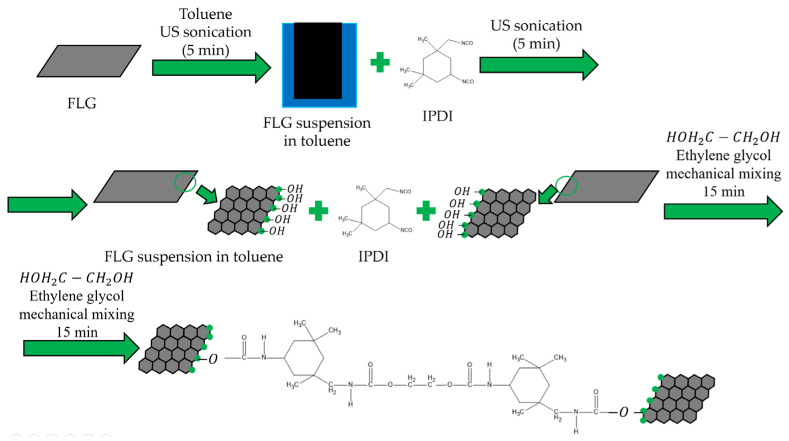
Synthesis scheme of coatings by chemical cross-linking of FLG particles.

A schematic diagram of the coating preparation process is presented in Figure 1.

Steel plates (grade St-08ps) with dimensions of 70 mm × 150 mm × 1.5 mm (length × width × thickness) were used as substrates. Isophorone diisocyanate (IPDI) (reagent grade, Sigma-Aldrich, Saint Louis, MO, USA) was used as the cross-linking agent, and ethylene glycol (reagent grade, LenReaktiv, St. Petersburg, Russia) was employed as the chain extender. IPDI is an aliphatic diisocyanate widely used as a curing agent in polymer science due to its reactive -NCO groups. The reaction scheme is presented in Figure 1.

For the coating preparation, a suspension of FLG particles in toluene (reagent grade, Sigma-Aldrich) was first prepared by ultrasonication for 5 min. IPDI was then added to this FLG suspension, followed by another 5-min ultrasonication step. Subsequently, a calculated amount of the chain extender (ethylene glycol) was introduced into the toluene-IPDI-FLG mixture. This final mixture was mechanically stirred at 200 rpm for 15 min.

**Figure 2:**
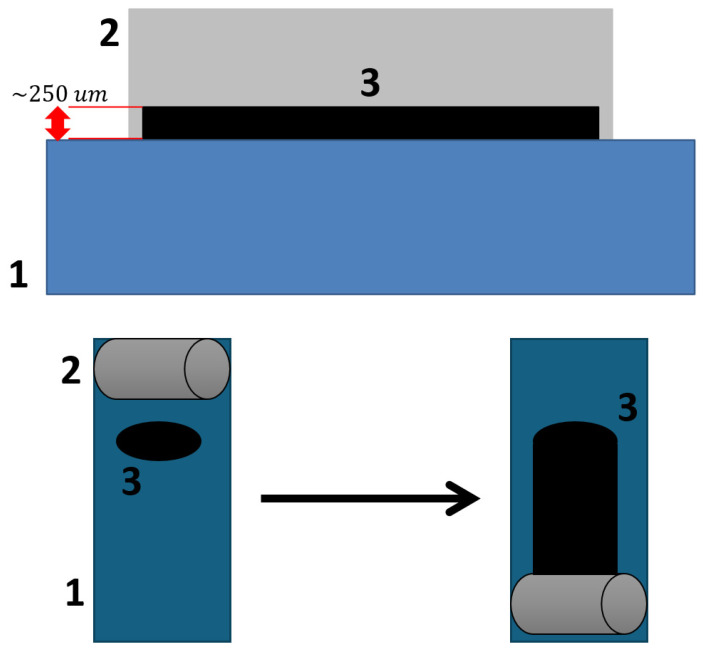
Principle of the applicator coating method.

**Figure 3:**
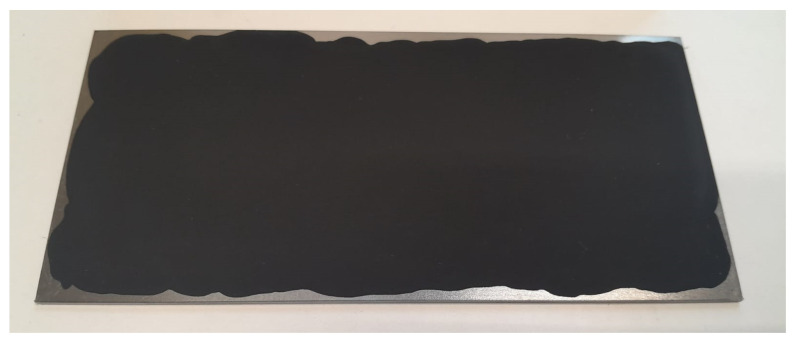
Photograph of the synthesized coatings based on FLG particles on a steel substrate.

The resulting suspension was applied to the steel substrate using an applicator with a steel roller, set to a 250 μm gap. The coating application principle is illustrated in Figure 2. After application, the coatings were cured in a drying oven at 100 °C for 24 h to remove residual toluene. The visual appearance of the obtained coatings is shown in Figure 3.

An epoxy resin-based coating was used as a reference. The system consisted of KER-828 resin (Kumho P&B Chemicals, Seoul, Republic of Korea), with an epoxide equivalent weight (EEW) of 0.53 mol/g, cured with a triethylenetetramine (TETA) hardener at a resin-to-hardener mass ratio of 10:1.

### 2.3. Characterization Methods for the Initial FLG and FLG-Based Coatings

The morphology of the FLG particles and the FLG-based coatings was examined using a Tescan Mira 3-M scanning electron microscope (SEM) (Brno, Czech Republic) equipped with an Oxford Instruments X-Max energy-dispersive X-ray spectroscopy (EDX) detector (Oxford, UK). The measurements were conducted at an accelerating voltage of 20 kV.

The particle size distribution of the FLG powder was determined by laser diffraction using a Mastersizer 2000 instrument (Malvern Panalytical, Malvern, UK), applying a disc-shaped particle model. For the analysis, a 0.05 wt.% suspension was prepared and dispersed in an ultrasonic bath for 5 min prior to measurement.

Fourier-transform infrared (FTIR) spectroscopy was performed using an Infralum FT-08 spectrometer (Lumex, St. Petersburg, Russia) in attenuated total reflectance (ATR) mode with a PIKE attachment (Pike Technologies, Madison, WI, USA).

Raman spectra were acquired using an Ntegra Spectra spectrometer (NT-MDT, Moscow, Russia) equipped with a 532 nm laser, a 100× objective, and a 600 grooves/mm grating. To avoid thermal effects, the laser power density was maintained below 50 µW/µm^2^. All measurements were performed at room temperature. Spectra were collected from several random points on each sample, and the presented data correspond to averaged spectra.

Coating thickness was measured using an RGK MC-25 micrometer (Russia). Adhesion to the steel substrate was evaluated according to the ASTM D3359 standard using a QFH-A cross-cut adhesion tester (China).

The water contact angle was determined by the sessile drop method using a 10 µL deionized water droplet. Images were captured with a digital camera, and the angle was calculated using the half-angle method.

The corrosion resistance of the coatings was tested in an HSL-60 T salt spray chamber (Guangdong, Dongguan, China) following the ASTM B117 standard. A 5 wt.% NaCl solution (pH adjusted to 6.5) was used as the corrosive medium. Prior to testing, cross-shaped scratches (100 mm in length, 0.4 mm in width) were made on the coated samples. The test duration was 48 h.

The electrical resistance of the FLG-based coating was measured using a Gigant GDM-3 multimeter (China).

## 3. Results

**Figure 4:**
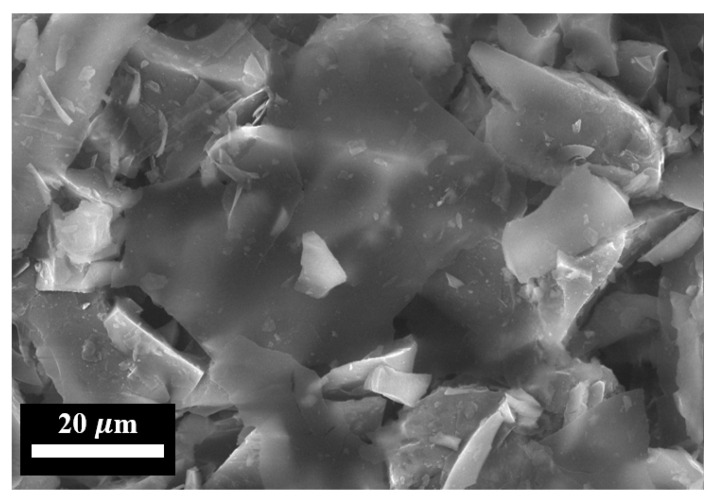
SEM image of initial FLG particles.

Figure 4 shows electron images of the FLG particles used for the synthesis of the coatings.

**Figure 5:**
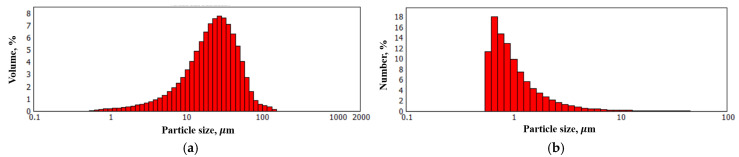
Particle size distribution of FLG. (**a**) Volume-based particle size distribution; (**b**) Number-based particle size distribution.

As shown in Figure 4, the synthesized FLG particles exhibit lateral dimensions on the order of several tens of micrometers. To obtain a quantitative size distribution, laser diffraction analysis was performed. The corresponding particle size distribution profile is presented in Figure 5.

As shown in Figure 5a, while particles with lateral dimensions up to several tens of micrometers are present in the FLG powder, their proportion is small. The majority of the particles, as indicated by the size distribution profile in Figure 5b, range from approximately 0.5 to 1 µm in size.

**Figure 6:**
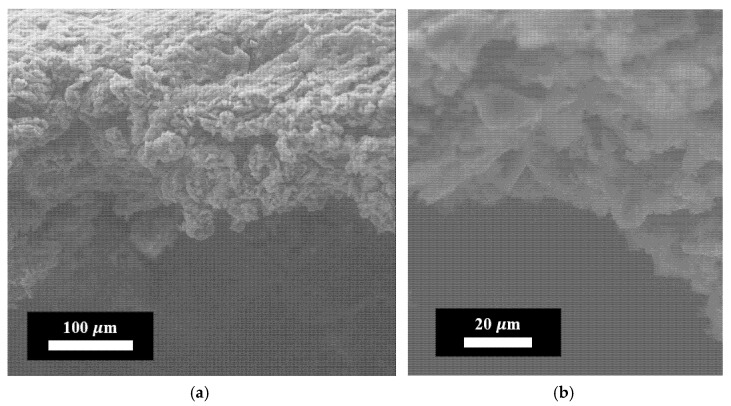
Electron images of the coating obtained by the chemical cross-linking method of FLG particles. (**a**) Linear scale = 100 μm; (**b**) Linear scale = 20 μm.

The average thickness of the epoxy reference coating was 240 ± 30 µm, comparable to that of the FLG-based coating at 250 ± 25 µm. To elucidate the internal structure of the FLG-based coating, scanning electron microscopy (SEM) analysis was performed. The resulting image (side view) of the coating, synthesized via chemical cross-linking of FLG particles, is presented in Figure 6.

As shown in Figure 6, the FLG-based coating exhibits a homogeneous macrostructure composed of randomly oriented FLG particles and their aggregates. This microstructure differs fundamentally from that of coatings produced by alternative methods, such as chemical vapor deposition (CVD), which typically yield continuous, aligned graphene films [35].

**Figure 7:**
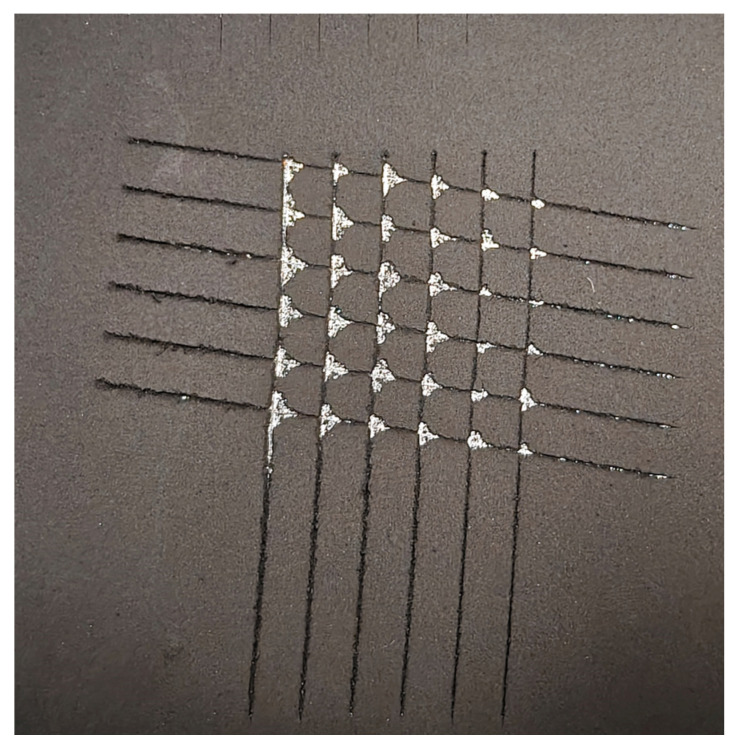
The results of the investigation into the adhesion strength of the MG-based coating.

The results of the adhesion strength tests for the synthesized coating are presented in Figure 7.

Adhesion testing, performed according to ASTM D3359, revealed localized delamination at the cut intersections. As shown in Figure 7, the detached area was less than 15%, which corresponds to a rating of 3A on the standard classification scale, indicating good adhesion.

**Figure 8:**
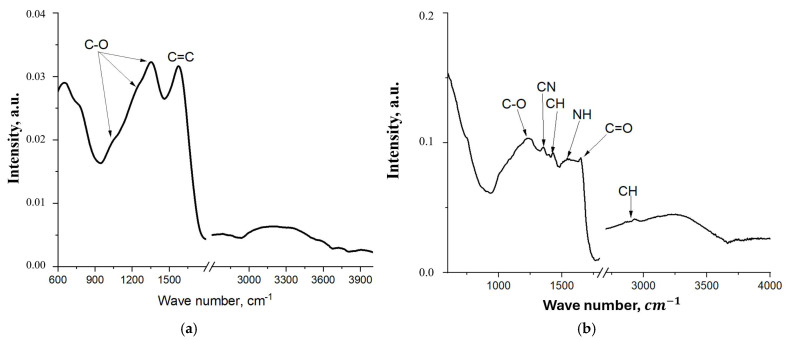
FT-IR spectrum of the initial FLG (**a**) coating obtained by the chemical cross-linking method of FLG particles (**b**).

The Fourier-transform infrared (FTIR) spectra of the initial FLG particles and the resulting cross-linked coating are compared in Figure 8.

The FT-IR spectrum of the pristine few-layer graphene (Figure 8a) exhibits two broad bands: one at 1224 cm^−1^, assigned to C–O stretching vibrations, and another at 1558 cm^−1^, attributed to C=C aromatic ring vibrations [36]. The significant broadening of these bands is likely due to the presence of a wide variety of alcohol and ether groups formed during the uncontrolled SHS process. In contrast, the spectrum of the cross-linked FLG-based coating (Figure 8b) displays several distinct vibrational bands corresponding to the newly formed chemical bonds. These include: C–O–C stretching at 1237 cm^−1^, C–N stretching in the urethane group at 1350 cm^−1^, C–H bending at 1430 cm^−1^, symmetric and asymmetric C–H stretching at 2865 cm^−1^ and 2931 cm^−1^, respectively, and N–H bending and C=O stretching (amide I) of the urethane group at 1530 cm^−1^ and 1650 cm^−1^ [37]. The emergence of these bands confirms the successful chemical cross-linking of the FLG particles. Notably, the intensities of the N–H and C=O bands are relatively weak, which can be attributed to their partial overlap with the broad C=C absorption band from the underlying graphene structure.

**Figure 9:**
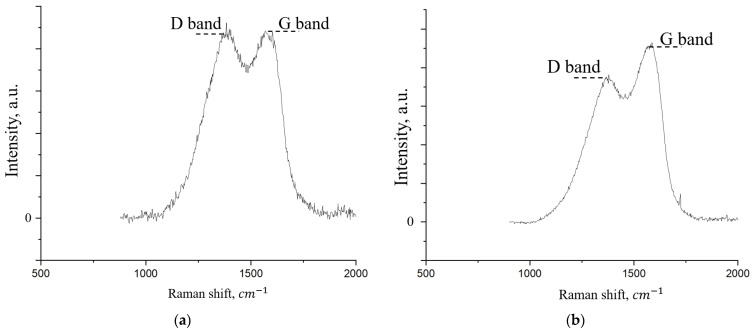
Raman spectrum of the initial FLG (**a**) and coating obtained by the chemical cross-linking method of FLG particles (**b**).

A comparison of the Raman spectra for the initial few-layer graphene (FLG) powder and the synthesized coating is presented in Figure 9.

As shown in Figure 9, the Raman spectra of both samples exhibit the characteristic D and G bands typical of graphene nanostructures [38]. The G band (~1580 cm^−1^) arises from the in-plane stretching vibrations of sp^2^-hybridized carbon atoms in the graphitic lattice. The D band (~1350 cm^−1^), in contrast, is associated with structural defects and disorders, including edges and functional groups [39]. A semi-quantitative analysis of the defect density can be performed using the intensity ratio of the D and G bands (I_D_/I_G_). The calculated I_D_/I_G_ ratio is 0.94 for the pristine FLG powder and decreases to 0.75 for the cross-linked coating. This reduction suggests a decrease in the overall defect density upon coating formation. A primary contributor to the D band intensity is the presence of graphene sheet edges where carbon bonds are terminated by functional groups such as -OH and -COOH [40]. Given that the coating is synthesized via chemical cross-linking reactions involving these very groups, we hypothesize that this process effectively “stitches” the edges, reducing the number of dangling bonds and thus leading to the observed decrease in the I_D_/I_G_ ratio.

**Figure 10:**
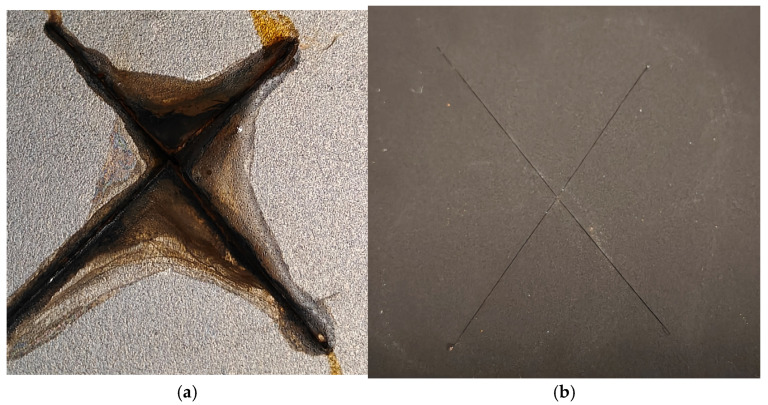
Images of the steel plates with the epoxy resin-based coating (**a**) and the FLG-based coating (**b**) after 48 h of exposure in the salt spray chamber.

Figure 10 shows the state of the coatings following a 48-h neutral salt spray test, revealing their comparative corrosion resistance.

**Figure 11:**
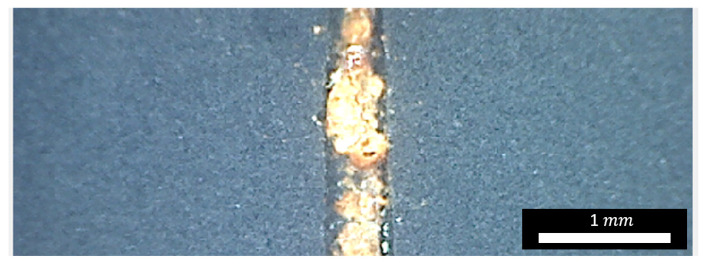
Image of the scratch area on the graphene nanoplatelet-based coating sample.

As shown in Figure 10a, significant corrosion and associated coating delamination are observed on the epoxy-based reference sample after 48 h of salt spray testing. In contrast, the sample with the FLG-based coating (Figure 10b) shows no signs of corrosion or delamination outside the pre-made scratch. The corrosion is confined solely to the scratch zone, as detailed in Figure 11.

**Figure 12:**
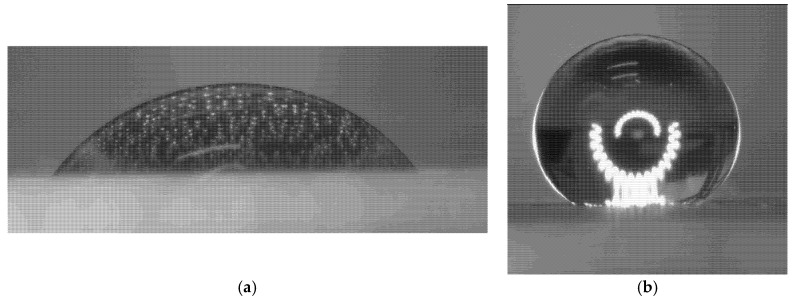
Images of water droplets on the epoxy-based coating (**a**) and on the graphene nanoplatelet-based coating (**b**).

The water contact angles of the coatings were measured to investigate the observed difference in corrosion resistance; the data are presented in Table 1 and Figure 12.

As shown in Table 1, the epoxy coating exhibits a moderately hydrophilic surface, whereas the FLG-based coating demonstrates pronounced hydrophobicity. This hydrophobic character likely arises from the chemical cross-linking of FLG particles via their surface -OH and -COOH groups, which reduces the number of free hydrophilic sites and creates a more non-polar surface.

The electrical resistance of the FLG-based coating was measured using a multimeter and was found to exceed 20 MΩ. This high resistance can be attributed to the specific microstructure of the coating. As shown in Figure 6, the coating consists of randomly oriented FLG particles and aggregates, creating a discontinuous, tortuous path for electron transport. Furthermore, while the synthesized FLG is free of Stone–Wales defects, as confirmed by Raman spectroscopy (Figure 9), it may contain other types of structural imperfections and edge terminations that contribute to the observed high resistivity.

**Figure 13:**
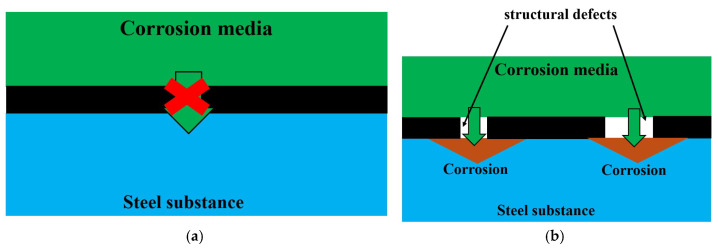
Influence of Defects on the Anti-Corrosion Efficacy of GNS-Based Coatings. Schematic of defect-free GNS (**a**) and GNS with defects (**b**).

A recent review [41] extensively discusses the parameters influencing the anti-corrosion efficacy of GNS-based coatings, highlighting two key challenges: (1) structural defects in GNS can serve as pathways for corrosive agents, and (2) the high intrinsic conductivity of pristine graphene can promote micro-galvanic corrosion at the coating-metal interface. This creates a significant dilemma, as common strategies to reduce conductivity (e.g., introducing defects) simultaneously compromise the barrier properties. The microstructure of our coating, characterized by high electrical resistance and the absence of extended conductive pathways, appears to mitigate both issues. A conceptual illustration of how defect-free, well-cross-linked GNS enhances barrier performance compared to defective or highly conductive structures is presented in Figure 13.

**Figure 14:**
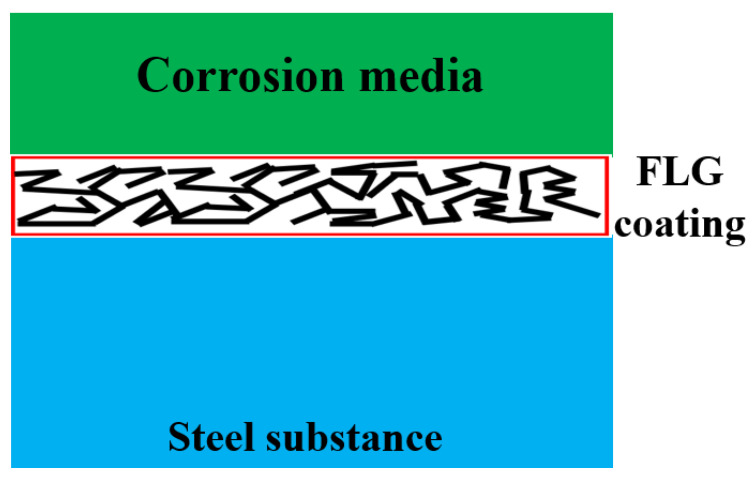
Proposed Anti-Corrosion Mechanism of the GNPs-Based Coating.

Based on our experimental results and existing theoretical models, we propose the following mechanism for the superior anti-corrosion performance of the synthesized FLG-based coating, as illustrated in Figure 14. The high efficacy stems from a synergistic effect of the unique properties of the SHS-synthesized FLG and the specific microstructure of the cross-linked coating.

First, the absence of Stone–Wales defects in the initial FLG [29] provides a fundamental advantage by reducing the intrinsic permeability of the individual graphene flakes to corrosive agents such as water and ions. Second, the coating exhibits high electrical resistance (>20 MΩ), which is attributed to its disordered, cross-linked microstructure and the presence of edge functional groups. This low conductivity effectively suppresses the formation of micro-galvanic couples at the graphene/metal interface, thereby mitigating a key corrosion pathway associated with conductive graphene materials. Finally, the protective architecture of the coating itself plays a crucial role. Composed of multiple, randomly oriented FLG particles and aggregates, it creates a highly tortuous path for any penetrating species. This overlapping effect means that even if a defect in one flake is bypassed, the corrosive medium is likely to encounter non-defective regions in overlapping flakes beneath, significantly hindering its progress toward the substrate. This multi-layered barrier compensates for the potential presence of other, non-Stone–Wales defects in individual flakes.

Thus, the anti-corrosion mechanism is not reliant on a single perfect property but on a strategic design that combines defect-minimized FLG with a non-conductive, overlapping layered structure to synergistically block corrosive penetration and eliminate galvanic corrosion pathways.

## 4. Conclusions

This study is the first to investigate the anti-corrosion efficiency of coatings based on few-layer graphene particles produced by chemical cross-linking.

It was found that these coatings are highly effective in protecting metals from corrosion, significantly outperforming standard epoxy resin coatings of similar thickness in neutral salt spray tests.

The high effectiveness of these coatings may be due to the absence of Stone-Wales defects in the few-layer graphene particles, as well as the overlapping effect provided by the presence of multiple graphene layers. This leads to a significant enhancement of the barrier effect, which determines the corrosion protection efficiency of coatings based on graphene nanostructures. Since the synthesis of few-layer graphene employs an original methodology based on the Self-propagating High-temperature Synthesis (SHS) process, it allows for the production of large material volumes at low cost, suggesting that the use of FLG-based coatings will be cost-effective.

To elucidate the possible mechanism behind the high anti-corrosion efficiency of such coatings, future work will involve comparative studies on the effectiveness of coatings made from chemically cross-linked graphene nanostructures with and without Stone-Wales defects. Another important direction for future research is the need for a quantitative assessment of the anti-corrosion performance of the coatings using electrochemical testing methods.

## Figures and Tables

**Table 1:** Results of thermal conductivity measurements of the samples.

Coating	Water Contact Angle
Epoxy	53 ± 3
FLG	136 ± 3

## Data Availability

Data are contained within the article.

## Abbreviations

The following abbreviations are used in this manuscript:

FLG	few-layer graphene
GNSs	graphene nanostructures
SHS	self-propagating high-temperature synthesis
